# Bridging the gap between feedstock growers and users: the study of a coppice poplar-based biorefinery

**DOI:** 10.1186/s13068-018-1079-y

**Published:** 2018-03-22

**Authors:** Chang Dou, Rick Gustafson, Renata Bura

**Affiliations:** School of Environmental and Forest Sciences, University of Washington, Box 352100, Seattle, WA 98195-2100 USA

**Keywords:** Biofuel system, Biorefinery, Feedstock plantation, Economic analysis, Integrated model, Poplar

## Abstract

**Background:**

In the biofuel industry, land productivity is important to feedstock growers and conversion process product yield is important to the biorefinery. The crop productivity, however, may not positively correlate with bioconversion yield. Therefore, it is important to evaluate sugar yield and biomass productivity. In this study, 2-year-old poplar trees harvested in the first coppice cycle, including one low-productivity hybrid and one high-productivity hybrid, were collected from two poplar tree farms. Through steam pretreatment and enzymatic hydrolysis, the bioconversion yields of low- and high-productivity poplar hybrids were compared for both sites.

**Results:**

The low-productivity hybrids had 9–19% higher sugar yields than the high-productivity hybrids, although they have the similar chemical composition. Economic calculations show the impact on the plantation and biorefinery of using the two feedstocks. Growing a high-productivity hybrid means the land owner would use 11–26% less land (which could be used for other crops) or collect $2.53–$3.46 MM/year extra revenue from the surplus feedstock. On the other side, the biorefinery would receive 5–10% additional revenue using the low-productivity hybrid.

**Conclusion:**

We propose a business model based on the integration of the plantation and the biorefinery. In this model, different feedstocks are assessed using a metric of product tonnage per unit land per year. Use of this new economic metric bridges the gap between feedstock growers and users to maximize the overall production efficiency.

**Electronic supplementary material:**

The online version of this article (10.1186/s13068-018-1079-y) contains supplementary material, which is available to authorized users.

## Background

Lignocellulose provides many opportunities to produce fuels and chemicals that are sustainable alternatives to production from current fossil resources. The success of lignocellulose-based products, however, is strongly dependent on having a cost competitive feedstock and high conversion yields.

Feedstock quantity plays a key role in the biofuels industry. Due to the relatively low-energy density of lignocellulosic biomass, a commercial-scale biorefinery requires a large feedstock supply [1, 2]. For feedstock growers, the goal is to increase the land productivity, maximize the biomass yield, and ultimately generate more profits. To address these issues, many studies have been done to develop fast growing energy crops using breeding and/or genetic tools [3, 4]. Higher productivity results in less land use and the feedstock growing, harvesting, and handling can be conducted in a smaller area to reduce the production cost.

Feedstock quality is critical to the conversion process. High feedstock quality is generally related to high sugar content, low lignin content, and ease of conversion, which leads to high fermentable sugar and final product yields [5]. Overall, the characterization of high-quality feedstock is dependent on the conversion yield per unit biomass. Upon receipt of harvested biomass, the feedstock users—the biorefineries—are most interested in the efficiency of biomass conversion. Sourcing a single type of feedstock with uniform composition year-round would be ideal to ensure a stable conversion process. In reality, feedstock quality varies depending on the crop variety, where it is grown, and how it is processed before reaching the biorefinery. Inconsistent feedstock quality will lead to fluctuations of the conversion yield [6–8]. Over the decades, substantial efforts have been made to improve the biomass conversion yield by optimizing process conditions for specific feedstocks [9–11]. Even for the same feedstock variety, however, heterogeneity of the biomass quality is prevalent, and the result is an uncertain final product yield. Several recent studies have shown that multiple physicochemical properties comprehensively affect the biomass recalcitrance and the subsequent sugar release and/or fuel production [12–15]. Those factors include, but not limit to, cellulose crystallinity and degree of polymerization, lignin content, lignin S/G ratio, and lignin molecular weight. Until now, there is no directly method to estimate the biomass recalcitrance or to evaluate the feedstock quality.

To expand the lignocellulosic bioeconomy, there is a need to treat lignocellulosic biomass as a fungible commodity [16, 17]. This would require the establishment of an efficient, large-capacity, and reliable supply system for producing and trading the lignocellulosic feedstocks [18]. Commodity goods are generally defined by their volume and standardized quality. However, unlike other commodities (e.g., corn grain, sugar, and lumber), biofuel feedstocks do not share a uniform format.

To reconcile the need for feedstock quantity in the supply chain and the need for feedstock quality in the conversion process, there must be a better understanding of feedstock from both sides. The overarching goal of the present work is to bridge the gap between the growers and users to facilitate future commoditization of lignocellulosic feedstock. This paper investigates the sugar yield in bioconversion of two poplar clones, one high-productivity hybrid and one low-productivity hybrid, from two coppice poplar plantation sites. With the plantation productivity data and bioconversion experimental results, we assessed the economics from the perspectives of feedstock growers and feedstock users. In addition, a new business model is introduced to account for the performance of both enterprises’ needs and to enhance the overall efficiency from feedstock production through biofuel conversion.

## Methods

### Research overview

The materials used in this research were 2-year-old short rotation coppice poplars harvested after the first rotation. Two hybrids—one with low-productivity and one with high-productivity—were collected from two planation sites (Jefferson, OR and Clarksburg, CA, USA). The previous research in our group showed that leaf material impedes bioconversion and lowers the overall sugar yield [19]. Therefore, all feedstocks in this study were leaf-free samples. After analyzing the chemical composition, four coppice poplar samples were processed through steam pretreatment at 195 °C for 5 min with SO_2_ (3% w/w) impregnation. The water-insoluble fractions (WIF) and water-soluble fractions (WSF) were separated and analyzed. The water-insoluble fraction solids were then enzymatically hydrolyzed at 5% (w/v) consistency and 5 filter paper units (FPU)/g cellulose enzymes loading. A complete mass balance was conducted to assess difference in sugar productions (in kg monomeric sugars/tonne biomass) of the four poplar samples. All experiments and analyses, including pretreatment, compositional analysis, enzymatic hydrolysis, and mass balance calculation, were run in triplicate.

From that, we estimated the corresponding ethanol production from each poplar feedstock. Economic analyses were made from both feedstock grower and user perspectives using the conversion results of the experiments and land productivity data of the tree farm. An evaluation was made to determine the impact of feedstock quality on the economics of commercial-scale cellulosic ethanol production. Given the land productivity (in tonne/acre/year) of each poplar feedstock, we compared the economics of feedstock growers using different hybrids on different plantation sites. Furthermore, the annual sugar yield per unit of land is calculated, leading to a new performance metric for the biofuels industry and providing a basis for commodity trading of lignocellulosic feedstocks.

### Raw material

Two poplar hybrids were studied in this research; a hybrid of *Populus trichocarpa* and *Populus deltoides* (the low-productivity hybrid) and a hybrid of *Populus deltoides* and *Populus maximowiczii* (the high-productivity hybrid). Both hybrids were cultivated in two plantation sites, one located in Jefferson, OR and one located in Clarksburg, CA. Poplars were established using a short rotation coppice management regime and harvested using a fully mechanized whole tree harvester. The samples used here were first rotation poplar trees harvested after their second growing season. Leaf materials negatively affect the bioconversion and were removed from the feedstock as described previously [19]. All samples were kept frozen at − 20 °C until use.

It should be noticed that the coppice poplar in this study is a new biofuel feedstock and has unique characteristics. According to the previous research in our group, the leaf-free biomass obtained from coppice poplar is a heterogenous mixture of juvenile wood, bark, and branches and is completely different from the regular poplar wood in many other studies [19, 20].

### Pretreatment and processing conditions

Four poplar samples were pretreated in the reaction conditions determined according to a previous study [21]. 600 g oven-dried (OD) weight of each feedstock was pre-impregnated overnight with anhydrous SO_2_ in plastic bags at atmospheric pressure. The amount of SO_2_ added to the bag corresponded to 3% (w/w) loading and was determined by weighing the bag before and after the addition of gas.

Steam explosion pretreatment was performed in a 2.7-l batch reactor (Aurora Technical, Savona, BC, Canada). Additional file 1: Figure S1 illustrates a schematic design of the reactor with a short description. Briefly, samples were loaded and heated at temperature 195 °C for 5 min. Following the reaction time, the pneumatic valve was opened to explode and discharge the biomass into a collection container. After steam explosion, the pretreated biomass slurry was separated into WSF and WIF using vacuum filtration. The WIF was then washed with a volume of deionized water equivalent to 20 times the dry weight of the sample to remove the free sugars.

### Enzymatic hydrolysis

Enzymatic hydrolysis was carried out using cellulase (Celluclast 1.5 l, Sigma-Aldrich Lot# 080M1599V) at 5 FPU/g cellulose (21.1 mg protein/g cellulose) and β-glucosidase (Novozyme 188, Sigma-Aldrich Batch# 097K0682) at 10 cellobiase units (CBU)/g cellulose (3.0 mg protein/g cellulose). The properties of enzymes are summarized in Additional file 1: Table S1. The WIF was hydrolyzed at 5% (w/v) consistency (solid loading) in a total volume of 50 ml in 125 ml Erlenmeyer flasks. The flasks were incubated at 50 °C and 175 rpm in a New Brunswick shaker. In addition, 50 mM citrate buffer was added to maintain the pH at 4.8, and tetracycline (40 µg/ml) and cycloheximide (30 µg/ml) were used to inhibit microbial contamination. 1 ml samples were taken periodically, boiled for 10 min to denature enzymes, filtered through a 0.22 µm syringe filter, and stored at − 20 °C until analysis.

### High pressure liquid chromatography (HPLC) analysis

The concentration of monomeric sugars from chemical composition analysis and enzymatic hydrolysis was measured on a Dionex (Sunnyvale, CA) HPLC (ICS-3000) system equipped with an AS autosampler, ED electrochemical detector, dual pumps, and anion exchange column (Dionex, CarboPac PA1). Deionized water at 1 ml/min was used as the mobile phase, and post-column addition of 0.2 M NaOH at a flow rate of 0.5 ml/min ensured optimization of baseline stability and detector sensitivity. After each analysis, the column was reconditioned with 0.2 M NaOH. Standards were prepared to encompass the same range of concentrations as the samples. Fucose was added to all samples and standards as an internal standard.

Acetic acid was measured using refractive index detection on a Shimadzu Prominence LC. Separation of these compounds was achieved by an anion exchange column (Rezex RHM Monosaccharide H^+^ (8%), Phenomenex, Inc., Torrance, CA) with an isocratic mobile phase that consisted of 5 mM H_2_SO_4_ at a flow rate of 0.6 ml/min. The column oven temperature was maintained at a constant temperature of 63 °C. Standards were prepared and used to quantify the unknown samples.

### Compositional analysis

#### Ash and extractives

Ash content of raw biomass samples was measured gravimetrically by heating 20-mesh-milled dry biomass to 575 ± 25 °C for 18 ± 6 h [22]. Water and ethanol extractives of raw biomass were determined according to National Renewable Energy Laboratory (NREL) methods [23].

#### Soluble fraction carbohydrates

Monomeric/oligomeric soluble carbohydrates were determined using NREL LAP TP-510-42623 [24]. Briefly, liquid samples were diluted by four times with water and 72% H_2_SO_4_ added to reach a pH of 0.07 (acid concentration of 4%). Samples were autoclaved at 121 °C for 60 min and analyzed by HPLC as described previously [25].

#### Insoluble fraction carbohydrates, acetate groups, and lignin

The chemical composition of raw biomass and WIF were determined according to a modified method derived from TAPPI Standard Method [26]. Briefly, 0.2 g of finely ground oven-dried sample is treated with 3 ml 72% H_2_SO_4_ for 120 min at room temperature, then diluted into 120 ml total volume and autoclaved at 121 °C for 60 min. Klason lignin contents were determined by gravimetric methods. After filtration through tared sintered-glass crucibles, the carbohydrate and acetyl composition of the filtrate is analyzed by HPLC and the acid soluble lignin in the filtrate is analyzed by UV at 205 nm.

### Sugar yield calculation

A complete mass balance was calculated using the composition and total mass of each WSF and WIF leaving pretreatment and enzymatic hydrolysis as described previously [21]. Glucose and xylose are the major sugars presented in the biomass and recovered during the process. Arabinose, galactose, and mannose were calculated as minor sugars. The sugar yield was defined as the total mass of monomeric sugars in the hydrolyzed solid and liquid phases normalized by the initial oven dry (OD) mass of biomass (kg monomeric sugars/tonne biomass).

### Economic modeling from feedstock growers and feedstock users

The economic potentials for growing and using different poplar hybrids were assessed from both the perspectives of feedstock growers and feedstock users. The annual feedstock processing capacity in the simulated cellulosic ethanol plant was set as 700,000 tonne. For the economic model of feedstock users, the annual ethanol production was calculated by implementing the sugar yield results in the current research and the fermentation conversion of NREL 2011 report [27]. The annual ethanol revenue was determined from the annual ethanol production at an ethanol selling price of $0.42/l ($1.59/gallon) [28]. These results were used to calculate the revenue differences associated with feedstock quality of different poplar hybrids.

For the economic model of feedstock growers, the average annual revenue was calculated based on the hybrid’s land productivity and feedstock price. The feedstock price ($53/tonne) was determined from the heating value of the poplar with the assumption that the only other realistic market for the first rotation 2-year-old poplar would be hog fuel [19]. A higher heating value (HHV) at 19.8 MJ/kg and a price of $2.8/MMBtu ($0.00265/MJ) were used for the calculation [19, 29]. In addition, given the feedstock processing capacity of the commercial-scale cellulosic ethanol plant and the land productivity of each poplar sample, the overall land requirement was calculated to indicate the impact of feedstock productivity on land usage.

The “land sugar productivity” is proposed in this paper to illustrate the plantation productivity with respect to biorefinery sugar production. Expressed as annual sugar yield per unit of land (kg sugars/acre/year), this metric is determined using the sugar conversion yield from the experimental data and the land productivity from the tree farm.

### Statistical analysis

The results were subjected to one-way analysis of variance (ANOVA) analysis followed by a Tukey’s test. All data are represented as the mean of triplicates with standard deviation. Chemical composition, sugar conversion of enzymatic hydrolysis, sugar yield following steam pretreatment, and enzymatic hydrolysis were analyzed based on 5% alpha level (95% confidence interval). Statistical differences in chemical composition and sugar yield were determined from *p* values (*p* < 0.05). Data were analyzed using R (version 3.0.1) software. In this manuscript, any data analysis stated as “significant” represents statistically significant (*p* value < 0.05).

## Results and discussion

### Bioconversion of coppice poplar for sugar production

#### Chemical composition of poplar feedstocks

Before bioconversion, all four feedstocks were characterized to determine the chemical composition. The compositional information is listed in Table 1. For the Jefferson site, the low-productivity hybrid had 1.7% lower total sugar content, 1.5% lower ash content, and 6.7% higher extractives content than the high-productivity hybrid. For the Clarksburg site, the chemical composition between two coppice samples were similar. The only significant differences were found in ash and extractives, where the low-productivity hybrid is 0.5 and 1.9% higher in ash and extractives than the high-productivity hybrid, respectively. Interestingly, both hybrids showed 2.4–3.5% higher sugar content in the Jefferson site than the Clarksburg site. Other constituents, however, did not show these differences between sites. Overall, the difference in the chemical composition, especially the sugar content and the lignin content, was minimal between the two hybrids in both sites.

**Table 1:** Chemical composition of low-productivity and high-productivity hybrids from two plantation sites (shown as weight percentage)

Site	Hybrid	Raw biomass composition (%)							
		Glucan	Xylan	Minor sugars	Total sugars	Lignin	Acetic acid	Ash	Extractives
Jefferson	Low-productivity	33.2	11.9	4.4	49.5	26.8	6.1	3.4	16.7
		0.5	0.3	0.1	0.2	1.0	0.0	0.0	0.0
	High-productivity	34.3	12.9	4.0	51.2	26.4	6.8	4.9	10.0
		0.2	0.1	0.1	0.3	1.8	0.1	0.5	0.7
Clarksburg	Low-productivity	36.6	12.3	4.1	53.0	27.0	6.2	3.2	16.4
		0.3	0.1	0.1	0.4	0.6	0.2	0.0	0.9
	High-productivity	37.5	12.5	3.6	53.6	27.5	6.2	2.7	14.5
		0.5	0.2	0.1	0.7	0.5	0.2	0.1	0.5

Standard deviations (SDs) are shown under each mean value

#### Chemical composition of water-insoluble fraction (WIF) and water-soluble fraction (WSF) after pretreatment

Following pretreatment and liquid–solid separation, the compositions of the water-insoluble fraction (WIF) and the water-soluble fraction (WSF) were analyzed. Expressed as percent of dry matter, Table 2 shows the WIF chemical composition. The sugar and lignin content ranged from 55.2 to 58.7% and from 36.8 to 37.4%, respectively, within the four coppice poplar samples. The trends of WIF sugar content were generally consistent with the raw biomass composition. Comparing between different hybrids of each site, the low-productivity hybrid comprised 1.3% less sugar and 1.5% more lignin than the high-productivity hybrid in Jefferson, while the low-productivity hybrid had 1.0% more sugar and 0.8% less lignin than the high-productivity hybrid in Clarksburg. The WIFs of Jefferson site had higher sugar content than those of Clarksburg site, which agrees with the sugar composition in raw biomass.

**Table 2:** Composition and sugar recovery resulting from steam pretreated of coppice samples including WIF yield (expressed as recovered solids in kg/tonne), chemical composition of WIF (as percentages of the solid weight), and monomeric sugar yields in WSF samples (expressed as kg/tonne raw biomass)

Site	Hybrid	WIF yield (kg/tonne)	Chemical composition of WIF (%)					Sugar recovered in WSF (kg/tonne)			
			Glucan	Xylan	Minor sugars	Total sugars	Lignin	Glucose	Xylose	Minor sugars	Total sugars
Jefferson	Low-productivity	468.5	52.2	2.4	0.7	55.2	37.4	49.4	78.2	28.7	156.3
		21.6	0.3	0.3	0.1	0.1	0.1	1.5	4.0	1.1	6.3
	High-productivity	467.4	53.0	2.8	0.7	56.5	35.9	27.2	65.7	20.5	113.3
		5.6	1.0	0.1	0.0	0.9	0.4	0.4	2.1	1.0	2.6
Clarksburg	Low-productivity	508.5	55.3	2.6	0.7	58.7	37.6	62.2	80.4	30.4	173.0
		9.3	0.6	0.1	0.0	0.5	0.5	2.6	3.9	0.4	6.1
	High-productivity	529.0	54.4	2.6	0.7	57.7	36.8	53.4	72.0	27.1	152.4
		6.4	0.4	0.2	0.0	0.2	0.7	4.0	4.6	2.5	11.1

Standard deviations (SDs) are shown under each mean value. kg/tonne values refer to kg of sugar recovered per tonne of raw OD biomass

Table 2 shows that the monomeric sugar yields in the WSF differ between poplar hybrids. As expected, the majority of minor sugars resided in the WSF since most hemicellulose was dissolved during pretreatment. Contrary to the small difference in WIF composition, a significant difference in WSF monomeric sugar yields was observed between different hybrids (Table 2). For samples from the Jefferson site, the yields of glucose, xylose, and minor sugar were 45, 16, and 29% higher in the low-productivity hybrid than the high-productivity hybrid, respectively. The difference was relatively smaller for samples from Clarksburg site. For Clarksburg, the yields of glucose, xylose, and minor sugar were 14, 10, and 11% higher in the low-productivity hybrid than the high-productivity hybrid, respectively.

#### Enzymatic hydrolysis of water-insoluble fraction (WIF)

**Fig. 1:**
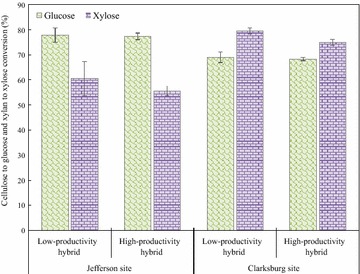
96 h cellulose to glucose and xylan to xylose conversion of water-insoluble fraction (WIF) of steam pretreated coppice poplar samples. Error bars indicate standard deviation from triplicates

The WIF of all samples were enzymatically hydrolyzed at 5% consistency with 5 FPU/g cellulose cellulase enzyme loading. Figure 1 shows the extent of cellulose and xylan conversion for the different hybrids. The cellulose and xylan conversion highlights the differences in hydrolyzability between different hybrids from the two sites. The maximum conversion was obtained after 96 h enzymatic hydrolysis for all the samples and these results were used to compare the hydrolysis of the different hybrids. For coppice poplar samples from the Jefferson site, there was no significant difference between hybrids; the cellulose to glucose conversion was 78–79% and xylan to xylose conversion was 58–60% for both the hybrids. For the two hybrids from Clarksburg, the glucan conversion was similar, while the low-productivity hybrid had higher xylan conversion (79%) than the high-productivity hybrid (75%). Interestingly, although there was no substantial difference in WIF hydrolyzability between hybrids, the cellulose and xylan conversions differed between sites. Poplar samples from the Jefferson had higher glucan but lower xylan conversion compared to samples from Clarksburg.

#### Overall sugar yield

**Fig. 2:**
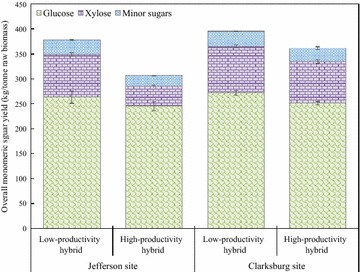
Overall sugar yield expressed as monomeric sugar per unit raw biomass (kg/tonne) of coppice poplar samples after pretreatment and enzymatic hydrolysis. Error bars indicate standard deviation from triplicates

The overall sugar yield was calculated based on the monomeric sugars present in the WSF and the monomeric sugars from hydrolyzed WIF. The overall sugar yield determines the total amount of fermentable sugars that can be obtained from each coppice poplar sample in the bioconversion process. Figure 2 presents the total sugar yield for two hybrids from two sites. The sugar yields ranged from 306 to 395 kg/tonne. For the Jefferson coppice poplar samples, 71 kg/tonne more monomeric sugars were recovered from the low-productivity hybrid compared to the high-productivity hybrid. For the Clarksburg coppice poplar samples, the overall monomeric sugar yield of the low-productivity hybrid was 395–34 kg/tonne higher than that of the high-productivity hybrid. Similar observations were described in the previous studies where transgenic poplars with higher growth efficiency had significant lower saccharification yields [30, 31].

It should be noticed that, contrary to the similar original sugar content in raw biomass, the overall sugar yields were substantially different between hybrids for both sites. As shown in Table 1, the differences in sugar composition of the raw biomass were negligible between hybrids in each site. Despite a slightly lower original sugar content, the low-productivity hybrid achieved 19% higher sugar yield compared to the high-productivity hybrid from the Jefferson site (Fig. 2). For the Clarksburg site, the hybrids had identical sugar content in raw biomass, but the sugar yield of the low-productivity hybrid was 9% higher than that of the high-productivity hybrid. The original sugar composition in raw biomass showed no correlation to the sugar yield. This highlights the difficulty in predicting the bioconversion yield when only given the compositional characteristics.

Biomass sugar content, especially the cellulose content, has been proposed as one of the prime targets to improve the feedstock quality using classic breeding and genetic transformation [32]. However, the findings in this study indicate that a higher sugar content in feedstock may not yield more sugar in bioconversion. Predicting the conversion yield by only knowing the feedstock chemical composition appears to be difficult. Recent research has shown that biomass recalcitrance is affected by multiple elements and cannot be simply judged on one factor [12–14, 33]. As such, test trials that simulate the conversion process are necessary to evaluate the process-oriented quality of lignocellulosic feedstock and potential product yields. Even for the same type of feedstock, trials are needed to better understand the processing performance of feedstock and to eliminate uncertainty from species variabilities [12, 13].

### Economics from growers and users vantage

The contrast between rapid growth and ease of conversion for the poplar hybrids discussed above motivated us to investigate the economic impacts of adopting specific hybrids for use in a biorefinery. Feedstock growers generally evaluate their economics based on the total biomass harvested from the plantation over a given year. Table 3 reveals the land productivity varies between hybrids and sites. The low-productivity hybrid delivered 26 and 11% less feedstock than the high-productivity hybrid at the Jefferson site and Clarksburg site, respectively. The difference between sites was more significant. For each hybrid, the Clarksburg site produced 43 and 53% less biomass than the Jefferson site.

**Table 3:** Plantation economics: including land feedstock productivity, land needed to meet the capacity of a commercial-scale biorefinery, and additional revenue of selling surplus feedstock

Site	Hybrid	Plantation economics			Biorefinery economics		
		Land feedstock productivity	Land needed^a^	Additional revenue^c^	Ethanol conversion yield	Annual ethanol production	Annual ethanol revenue
		(tonne/acre/year)	(MM acre/year)^b^	($MM/year)	(liter/tonne)	(MM liter/year)	($MM/year)
Jefferson	Low-productivity	2.78	0.25	–	214	150	63
	High-productivity	3.75	0.19	3.46	179	125	53
Clarksburg	Low-productivity	1.59	0.44	–	223	156	66
	High-productivity	1.78	0.39	2.53	205	143	60

Biorefinery economics: including conversion efficiency, annual ethanol production, and annual ethanol revenue using different poplar hybrids from two sites

^a^Land area required to meet the biorefinery with feedstock capacity of 700,000 dry tonne/year

^b^MM stands for one million

^c^Feedstock grower additional revenue calculated based on selling surplus feedstock at the price of $53/tonne [19]

The land area required to produce feedstock for the biorefinery is highly dependent on the crop productivity. By assuming one feedstock grower provides all the feedstocks for a biorefinery that processes 700,000 tonne biomass each year, we calculated the land area needed to meet the feedstock supply for each hybrid grown at each site. As shown in Table 3, the differences were significant. At the Jefferson site, growing the high-productivity hybrid requires 0.65 MM acres less land than the low-productivity hybrid, which represents an over 25% reduction in land requirement. At the Clarksburg site, selecting the high-productivity hybrid could save 0.48 MM acres (11%) of the land area.

Alternatively, switching from a low-productivity to a high-productivity hybrid would yield more feedstock on a given land base and more revenue for the farmer. If the extra feedstock obtained by switching hybrids is sold as hog fuel at price of $53/tonne [19], replacing the low-productivity hybrid by the high-productivity one could result in a $2.53–$3.46 MM/year additional revenue (Table 3). Of note, even modest improvements in land productivity will dramatically reduce the land area requirement or generate additional revenue. Taken together, the difference in productivity is substantial and will be a key factor for feedstock growers to consider in selecting which clone to plant.

The economic perspective of the feedstock user, who is mostly concerned with conversion yields, is different than that of the feedstock grower. Experimental data from this study was applied to economic models to investigate the impact of feedstock quality on ethanol production in a commercial-scale cellulosic biorefinery. We compared the revenue difference in selling ethanol as the final product using different coppice poplars as biorefinery feedstocks. The annual feedstock processing capacity of the simulated biorefinery was set as 700,000 tonne. As shown in Table 3, the ethanol conversion yield ranged from 179 to 223 l/tonne, and the corresponding biorefinery ethanol production of varied from 125 to 156 MM l/year. The annual ethanol production using the low-productivity hybrid was 16% and 8% higher than the high-productivity hybrid for the two sites. Given the ethanol price $0.42/l ($1.59/gallon) [28], the annual revenue of a biorefinery using low-productivity hybrid as feedstock was $ 10 and $ 5 MM higher than a biorefinery using high-productivity hybrid from the Jefferson site and Clarksburg site, respectively.

### An overarching performance metric

To date, no standard has been established for the quality of lignocellulosic feedstocks. Quality standards will be required, however, for lignocellulosic biomass feedstocks to be traded as commodity products.

The current lignocellulosic feedstocks trading is based on the dry weight of biomass. Our findings show that if the feedstock conversion facilities do not consider quality variations inherent in the feedstocks, they may experience considerable fluctuations in bioconversion product yield. Even knowing the composition of the feedstock, it might still be risky to estimate the final product yield just based on the sugar content in the raw biomass. From this study, we suggest that biorefineries have to appreciate the quality variations of feedstocks and pay attention to the inconsistencies in final product yields.

Given that different hybrids will have different productivity and different conversion yields, it would be useful to have an overarching metric that accounts for both of these important economic drivers. A metric of product volume (or mass) per acre per year combines the effects of land productivity and conversion yield. Table 4 shows the annual sugar productivity and ethanol output per unit of land between different hybrids and sites. It gives a completely different view from the information provided in Table 3. For every acre of land in Jefferson site, the high-productivity hybrid could obtain 8.7% higher sugar production and 11% higher ethanol output than the low-productivity hybrid. Given an ethanol selling price of $0.42/l ($1.59/gallon), that means the land revenue of the high-productivity hybrid is $32 per acre per year higher than the low-productivity hybrid in Jefferson site. The difference between hybrids in Clarksburg site was smaller − 2.4% for sugar productivity and 3% for ethanol output, which corresponds to a land revenue difference of $4 per acre per year. Comparing the productivity of the two sites, the ethanol output from an acre of the Jefferson site would be 40–46% higher than that from the Clarksburg site.

**Table 4:** Land productivity in terms of sugar yield, ethanol output, and revenue

Site	Hybrid	Integrated model economics		
		Land sugar productivity	Land ethanol output^a^	Land revenue
		(tonne/acre/year)	(liter/acre/year)	($/acre/year)
Jefferson	Low-productivity	1.05	594	250
	High-productivity	1.15	670	282
Clarksburg	Low-productivity	0.63	355	149
	High-productivity	0.64	365	153

^a^ Fermentation conversion calculated based on NREL 2011 biochemical conversion report [27]

Reconciling feedstock growers and users will be challenging given the current status of the lignocellulosic supply chain and biorefineries. A business model that integrates feedstock plantations and biorefineries would ultimately solve this problem and drive the industry to an overall greater productivity. This strategy is being applied today for different types of crops, including sugarcane and pulpwood in South American and oil palm in Southeast Asia. For example, Klabin—the Brazilian paper manufacturer—has its own nursery, plantation, and pulp mill [34] and uses a metric of tonnes of pulp per hectare per year to assess their overall pulping performance [35]. This allows the company to breed and choose the most productivity crop with the best pulp quality. A similar integrated approach in the biofuels industry could enable a more holistic approach to developing the industry.

## Conclusions

The quality of 2-year-old coppice poplar varies between hybrids and plantations, leading to different product yields in biochemical conversion. Although having similar sugar contents, the low-productivity hybrid showed 9–19% higher sugar yield compared to the high-productivity hybrid from two sites. Selection of hybrids can significantly impact the economics for the feedstock users. However, the economics of feedstock growers are largely driven by the land productivity. The definition of commodity-type lignocellulosic feedstock could be reconsidered: not the dry weight of raw biomass or total sugar in the raw biomass, but the total amount of sugars that can be obtained in the conversion process. By evaluating the feedstock quality that accounts for bioconversion yield, a more reasonable pricing strategy may be useful. A metric that combines the plantation productivity and bioconversion yield would provide an overarching measure of performance. An integrated business model with the plantation being economically tied to the biorefinery would eliminate differences between feedstock growers and users and would improve the overall efficiency of biofuel production.

## Additional file

**Additional file 1: Figure S1.** Schematic diagram of the bench-scale steam explosion reactor. **Table S1.** Properties of enzymes applied in hydrolysis.

## Abbreviations

MM: one million
WSF: water-soluble fraction
WIF: water-insoluble fraction
OD: oven dry
CBU: cellobiase unit
HPLC: high-pressure liquid chromatography
NREL: National Renewable Energy Laboratory
ANOVA: analysis of variance
HHV: higher heating value
