# Supplementary material for: Bridging the gap between feedstock growers and users: the study of a coppice poplar-based biorefinery
Source: Biotechnol Biofuels. 2018 Mar 22;11:77. doi: 10.1186/s13068-018-1079-y (PMC5863363; doi:10.1186/s13068-018-1079-y)
Supplement: Supplementary file 1 — Additional file 1: Figure S1. Schematic diagram of the bench-scale steam explosion reactor. Table S1. Properties of enzymes applied in hydrolysis. [file 13068_2018_1079_MOESM1_ESM.docx]

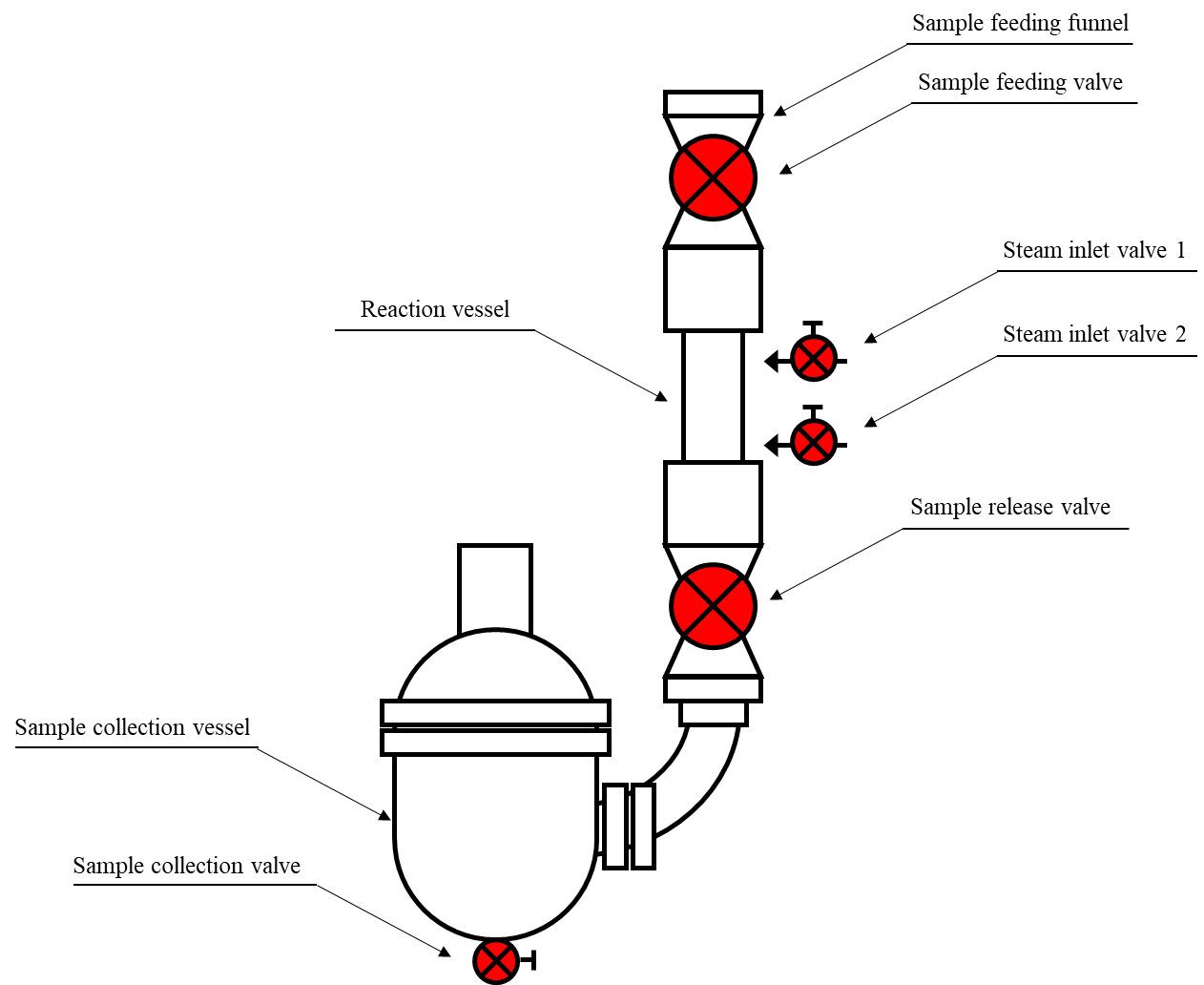


Figure S1 Schematic diagram of the bench-scale steam explosion reactor.

The reactor was preheated to pretreatment temperature prior to the experiment. Biomass samples were then fed into the reactor vessel through the sample feeding funnel and valve. Upon closure of the sample feeding valve, the steam was injected into the reactor vessel to heat up and maintain the reaction temperature. It takes about 30s to heat up of the reactor vessel holding 300 g OD poplar samples to 195 °C. After 5 mins elapsed, the sample release valve opened. The pretreated biomass was ejected into a sealed sample collection vessel coated with cooling water. The biomass was chilled to room temperature within 1 min. Finally, the pretreated biomass was collected from the collection vessel.

Table S1. Properties of enzymes applied in hydrolysis.

| Enzymes | Protein content | Cellulase | β-glucosidase |
| --- | --- | --- | --- |
|  | (mg/ml) | (FPU/ml) | (U/ml) |
| Celluclast 1.5 L | 109.8 | 26.0 | - |
| Novozyme 188 | 148.3 | - | 492.0 |
